# The Protective Effect of Traditional Chinese Medicine on Liver Ischemia-Reperfusion Injury

**DOI:** 10.1155/2021/5564401

**Published:** 2021-04-08

**Authors:** Wen Ma, Songling Tang, Dina Xie, Guoqiang Gu, Lu Gan

**Affiliations:** ^1^Sichuan University-The Hong Kong Polytechnic University Institute for Disaster Management and Reconstruction, Chengdu 610207, China; ^2^Research Laboratory of Emergency Medicine, Department of Emergency Medicine, West China Hospital, Sichuan University, Chengdu 610041, China; ^3^Department of Emergency Medicine and Medicine, Thomas Jefferson University, Philadelphia 19107, PA, USA; ^4^Cardiology Department, Second Hospital of Hebei Medical University, Shijiazhuang 050000, Hebei, China; ^5^National Clinical Research Center for Geriatrics, West China Hospital, Sichuan University, Chengdu 610041, China

## Abstract

Liver ischemia-reperfusion (I/R) injury occurs during transplantation and major hepatic surgery, which may lead to postoperative liver dysfunction. More and more traditional Chinese medicines (TCMs) have been used to treat liver ischemia-reperfusion injury. The purpose of this review is to evaluate the different protective effects of TCMs in the treatment of liver ischemia-reperfusion injury and to summarize its possible mechanisms. The results indicate that TCMs attenuate liver I/R injury via multiple mechanisms, including antioxidation stress, anti-inflammatory response, antiapoptosis, and inhibiting endoplasmic reticulum stress. However, the in-depth mechanism of the protective effects of these traditional Chinese medicines still remains unknown.

## 1. Introduction

Ischemia-reperfusion (I/R) injury is a two-stage phenomenon in which blood flow into the organ is reduced, leading to tissue hypoxia and cell damage, followed by aggravated injury when oxygen delivery is restored [1]. Liver ischemia-reperfusion injury (I/RI) occurs during transplantation and major hepatic surgery, which may result in postoperative liver dysfunction [2, 3]. The cessation of blood flow to an organ can lead to oxygen and nutrient deficiencies which can induce inflammatory cell infiltration, production of oxygen-derived reactive oxygen species (ROS) and nitrogen-derived reactive nitrogen species, and process during the reperfusion period [4]. Reperfusion injury mainly comes from toxic ROS produced by ischemic tissue when oxygen is reintroduced. ROS can be derived intracellularly and extracellularly, and mitochondria in liver cells are the main source of ROS [5].

Herbal medicine has drawn more and more attention in recent years. According to the World Health Organization, approximately 80% of the global population relies on traditional herbal medicines as part of standard health care [6]. A series of traditional Chinese medicine ingredients have been used to treat liver ischemia-reperfusion injury and have achieved good results (see Table 1). However, traditional Chinese medicine is a multicomponent medicine and plays an effective role through multiple targets and pathways, including antioxidation stress, anti-inflammatory response, antiapoptosis, and inhibiting endoplasmic reticulum stress. In this review, we summarize the effects of TCMs on I/R-induced liver injury, with focus on the possible underlying mechanisms.

## 2. The Protective Effect of TCMs on Liver Ischemia-Reperfusion Injury and Potential Mechanisms

During I/R, some functional changes that occur at the cellular level may cause cell damage via production of ROS, inflammatory cytokines, and chemokines. These events trigger the apoptotic pathway and ultimately lead to organ failure [24]. According to current researches, the protective effect of TCMs on liver I/R is mainly involved in several mechanisms: antioxidation stress, anti-inflammatory response, antiapoptosis, and inhibiting endoplasmic reticulum stress. Also, there are two main models used in animal experiments: the hepatic I/RI model and liver transplantation model.

### 2.1. Antioxidative Stress

It is well known that oxidative stress and reactive oxygen intermediates play important roles in liver ischemia-reperfusion injury. Free radicals formed by oxidative stress damage the cell membrane of hepatocytes through lipid peroxidation or/and other means. Furthermore, these free radicals can cause extensive damage to DNA and proteins, which can eventually lead to acute and chronic liver damage [25–27]. Lots of research studies are focused on antioxidant compounds extracted from herbal medicines to address the mechanism of its clinical protective effect to liver I/R injury.

In I/RI model rats, *Atractylodes macrocephala* polysaccharide (AMP), the principal bioactive component of *Atractylodes macrocephala*, significantly inhibited lipid peroxidation and altered the activities of the antioxidant enzyme, superoxide dismutase, and malondialdehyde level, which is associated with its antioxidant properties and inhibition of NF-*κ*B activation [7]. Saffron ethanol extract (SEE) contains abundant flavonoid compounds with antioxidant effect [28]. A study found that SEE could reduce liver IR damage by scavenging free radicals, maintaining physiological ROS level, and attenuating oxidation-mediated chaperone carbonylation [8]. Mitochondria are the main source of ROS in cells. Mitochondrial damage leads to an increase in ROS production, which results in oxidative stress [29]. Mitofusin 2 (Mfn2), located in the outer mitochondrial membrane, has the function of controlling mitochondrial metabolism [30]. Lou et al. found that breviscapine, a flavonoid compound extracted from the natural plant *Erigeron breviscapus* [31], could attenuate liver I/R injury by reducing lipid peroxidation and downregulating the expression of Mfn2 via inhibiting the Ras-PI3K-Akt pathway [9]. Caffeic acid (CA), a single phenolic acid derived from *Salvia miltiorrhiza* [32], is associated with chondriosome. It was found to have a protective effect on I/RI by reducing liver microcirculation disturbance and oxidative damage through regulating Sirt3 and the mitochondrial respiratory chain [10]. Further research shows that PDIA3 (protein disulfide isomerase A3) activates NADPH oxidase and causes the burst of ROS. CA may protect the transplanted liver by inhibiting PDIA3-NADPH oxidase [11]. TCMs exert hepatic ischemia-reperfusion injury protection through antioxidative stress which is also observed in huperzine A (HupA), gypenoside (GP), and glycyrrhizin (GL) [14–16].

### 2.2. Anti-Inflammatory Response

The liver undergoes a strong inflammatory process during ischemia and reperfusion injury. This liver inflammation is initially triggered by ischemia. However, the inflammation mainly occurs during the reperfusion phase and is characterized by the recruitment of large numbers of neutrophils in the liver. The production of cytokines, chemokines, and danger signals activates resident liver cells, white blood cells, and Kupffer cells [33]. The following research studies have authenticated that TCMs attenuate liver ischemia and reperfusion injury through an anti-inflammatory response pathway.

Ischemia reperfusion is considered to be a complex cascade of inflammatory mediators involved in the pathogenesis of liver injury. Different from caffeic acid, although magnesium lithospermate B (MLB) and Tanshinone IIA (Tan IIA) are also the main components of *Salvia miltiorrhiza*, they mainly exert anti-inflammatory effects. MLB can prevent the activation of inflammatory signaling pathways, reduce the expression of inflammatory mediators, and decrease the infiltration of macrophages and neutrophils, thereby reducing the damage of liver cells induced by IR [12]. It was reported that IL-17 contributes to the accumulation of neutrophils in the inflammatory liver. Triptolide, a purified ingredient of shrub-like vine *Tripterygium wilfondii* Hook F, can reduce the expression of IL-17 by inhibiting transcription 3 (STAT3) phosphorylation, thereby inhibiting the recruitment of neutrophils in the process of liver I/R [17]. In addition, there is evidence that Tan IIA pretreatment can reduce inflammation infiltration and liver damage. The underlying mechanism may be that Tan IIA inhibits the Toll-like receptors 4 (TLR4) signaling pathway, thereby enhancing the expression of HO-1 and reducing the expression of liver proinflammatory cytokines [13]. In Xiao's experiment, puerarin nanoparticle synthesis significantly decreased the TLR4 and NF-*κ*B expressions, which showed that puerarin can display its protective role by restraining the activation of proinflammatory factors through the TLR4/NF-*κ*B fashion [18].

Proinflammatory cytokines such as TNF-*α* and IL-6 play a key role in liver I/R injury. It has been found that levo-tetrahydropalmatine (L-THP), an active component of *Corydalis yanhusuo*, can inhibit the release of TNF-*α* and IL-6 induced by liver I/R, and this protective effect is partly dependent on the inhibition of the TNF-*α*-mediated ERK/NF-*κ*B pathway [19]. Similarly, astragaloside IV (AS-IV), a small molecular saponin, protects liver against ischemia-reperfusion injury by inhibiting the activation of NF-*κ*B in the reperfusion phase and reducing TNF-*α* [20]. Liu et al. found that Xuebijing (XBJ) with protective function of liver I/RI is largely due to its direct effect on the activation of hepatocyte inflammasomes and caspase-1-dependent IL-1*β* production, in addition to affecting the production of inflammatory factors/chemokines by Kupffer cells through NF-*κ*b-dependent mechanisms [21].

### 2.3. Antiapoptosis

Oxidative stress and/or mitochondrial dysfunction induced by hepatic ischemia reperfusion can eventually activate apoptotic cascade. Caspase-3 and -8 are key members of the cysteine-aspartate-specific protease family and have been shown to be essential for apoptosis [34]. Hepatocyte apoptosis is one of the most important cell death types in the process of liver I/R injury [35]. The activation of caspase-3 and caspase-8 was found in various apoptotic cells [36], and the upregulation of caspase-3 and caspase-8 was also found in I/R-induced liver injury, indicating that caspase-mediated apoptosis is essensial in organ I/R injury [37]. In addition to the caspase pathway, Bcl-2 family proteins also play a key role in the regulation of neuronal apoptosis. The following studies have demonstrated that TCMs have an effective performance in hepatic ischemic reperfusion injury through the antiapoptosis pathway.

Moreover, HupA, an alkaloid extracted from *Huperzia serrata*, can reduce liver I/R damage by reducing the expression of apoptosis-related proteins caspase-3, Bcl-2, and Bax [14]. The antiapoptotic effect of GP, the main ingredient of *Gynostemma pentaphyllum*, is related to the inhibition of I/R-induced increase in the activities of proapoptotic proteins Bax, cytochrome c, and caspase-3/8, as well as the decrease in the level of antiapoptotic protein Bcl-2 [15]. In Wang's study, it was shown that the *Ginkgo biloba* Dropping Pill (GBDP) can inhibit the expression of apoptosis-related protein markers in vitro. Consistent with the results of in vitro experiments, animal experiments confirmed that GBDP can downregulate the expression of proapoptotic proteins and reduce hepatocyte apoptosis caused by liver I/R injury [22].

### 2.4. Inhibition of Endoplasmic Reticulum Stress

Endoplasmic reticulum (ER) stress refers to the continuous accumulation of misfolded or unfolded proteins in the endoplasmic reticulum lumen, which activate the unfolded protein response (UPR) under pathological conditions. In a steatotic liver, endoplasmic reticulum stress is considered to be the main cause of posttransplant injury [38].

Zhang et al. found that berberine (BBR), a compound derived from the traditional Chinese medicine plants, inhibits endoplasmic reticulum stress-mediated phagocytosis in steatotic liver transplantation. It is the first report that addresses the protective function of BBR on steatosis liver transplantation, but the specific mechanism involved still remains unclear [23]. Besides, saffron ethanol extract can also relieve the endoplasmic reticulum stress and protein ubiquitination induced by liver I/R [8].

## 3. Conclusions and Prospects

Through this review, we get some similarities from articles published in recent years. Firstly, traditional Chinese medicines with protective effect of liver I/R injury are mostly the main active substances of Chinese medicines. Secondly, TCMs protect liver function via multiple mechanisms, including antioxidation, anti-inflammatory, inhibition of cell apoptosis, and inhibition of endoplasmic reticulum stress, of which antioxidant and anti-inflammatory effects are most commonly reported. Also, the NF-*κ*B signaling pathway is the most frequently involved signaling pathway. Thirdly, different components of traditional Chinese medicines may exert protective effects through different mechanisms. Lastly, the specific signaling pathways involved in these mechanisms remain unkown.

In conclusion, traditional Chinese medicine has protective effect on liver I/R injury. However, future research should pay more attention to in-depth mechanism exploration rather than just descriptive observations. Moreover, research should also clarify which components of Chinese medicine mainly play a protective role in liver ischemia reperfusion. Furthermore, it is necessary to conduct experiments both in vivo and in vitro to increase the convincing power of the experimental results. Finally, large sample, randomized, double-blind, placebo-controlled, and multicenter clinical trials are still in need.

## Figures and Tables

**Table 1:** The protective effect of TCMs on liver ischemia-reperfusion injury and potential mechanisms.

Traditional Chinese medicine	Major active ingredients	Models	Animals	Protective effects	Potential mechanisms	Ref.
*Atractylodes macrocephala*	*Atractylodes macrocephala* polysaccharide (AMP)	Hepatic I/RI model	SD rats	Antioxidation stress	NF-*κ*B signaling pathway	Jin et al. [7]
Saffron	Saffron ethanol extract (SEE)	Hepatic I/RI model	Wistar rats	Antioxidation stress; inhibition of endoplasmic reticulum stress	—	Pan et al. [8]
Breviscapus	Breviscapine	Hepatic I/RI model	SD rats	Antioxidation stress	Mfn2/Ras-PI3K-Akt pathway	Lin et al. [9]
*Salvia miltiorrhiza*	Caffeic acid (CA)/3, 4-dihydroxycinnamic acid	Hepatic I/RI model	SD rats	Antioxidation stress; Anti-inflammatory response	Sirt3 signaling pathway	Mu et al. [10]
	Caffeic acid (CA)/3, 4-dihydroxycinnamic acid	Liver transplantation model	SD rats, hepatocyte	Antioxidation stress; anti-inflammatory response	PDIA3-NADPH signaling pathway	Mu et al. [11]
	Magnesium lithospermate B (MLB)	Hepatic I/RI model	C57BL/6 mice	Anti-inflammatory response	NF-*κ*B signaling pathway	Song et al. [12]
	Tanshinone IIA (Tan IIA)	Hepatic I/RI model	C57BL/6 mice	Anti-inflammatory response	TLR4 signaling pathway	Qi et al. [13]
*Huperzia serrata*	Huperzine A (HupA)	Hepatic I/RI model	Wistar rats	Antioxidation stress; antiapoptosis	—	Xu and Wang [14]
*Gynostemma pentaphyllum*	Gypenoside (GP)	Hepatic I/RI model	C57BL/6 mice	Antioxidation stress; antiapoptosis	—	Zhao et al. [15]
*Glycyrrhiza uralensis*	Glycyrrhizin (GL)/Glycyrrhizic acid	Hepatic I/RI model	SD rats	Antioxidation stress	Nrf2/HO-1 signaling pathway	Kou et al. [16]
*Tripterygium wilfondii* Hook F	Triptolide (diterpenoid triepoxide)	Hepatic I/RI model	C57BL/6 mice, splenocytes	Anti-inflammatory response	STAT3 signaling pathway	Wu et al. [17]
Kudzu	Puerarin/7, 4-dihydroxyisoflavone-8*β*-glucopyranoside	Hepatic I/RI model	SD rats	Anti-inflammatory response	TLR4/NF-*κ*B pathway	Xiao et al. [18]
*Corydalis yanhusuo*	Levo-tetrahydropalmatine (L-THP)	Hepatic I/RI model	BALB/*c* mice	Anti-inflammatory response; antiapoptosis	ERK/NF-*κ*B pathway	Yu et al. [19]
*Astragalus membranaceus*	Astragaloside IV (AST-IV)	Liver transplantation model	SD rats	Anti-inflammatory response	NF-*κ*B signaling pathway	Chen et al. [20]
Chinese medicine mixture	Xuebijing (XBJ)	Hepatic I/RI model	C57BL/6 mice	Anti-inflammatory response	NF-*κ*B signaling pathway	Liu et al. [21]
*Ginkgo biloba* leaf	*Ginkgo biloba* Dropping Pill (GBDP)	Hepatic I/RI model	C57BL/6 mice, hepatocytes	Antiapoptosis	—	Wang et al. [22]
Chinese medicine mixture	Berberine	Liver transplantation model	Wistar rats	Inhibiting endoplasmic reticulum stress	—	Zhang et al. [23]
